# Cephalopod Behavior: From Neural Plasticity to Consciousness

**DOI:** 10.3389/fnsys.2021.787139

**Published:** 2022-04-12

**Authors:** Giovanna Ponte, Cinzia Chiandetti, David B. Edelman, Pamela Imperadore, Eleonora Maria Pieroni, Graziano Fiorito

**Affiliations:** ^1^Department of Biology and Evolution of Marine Organisms, Stazione Zoologica Anton Dohrn, Naples, Italy; ^2^Department of Life Sciences, University of Trieste, Trieste, Italy; ^3^Department of Psychological and Brain Sciences, Dartmouth College, Hanover, NH, United States; ^4^Association for Cephalopod Research ‘CephRes' a non-profit Organization, Naples, Italy

**Keywords:** cephalopods, behavioral plasticity, cognition, consciousness, neural plasticity

## Abstract

It is only in recent decades that subjective experience - or consciousness - has become a legitimate object of scientific inquiry. As such, it represents perhaps the greatest challenge facing neuroscience today. Subsumed within this challenge is the study of subjective experience in non-human animals: a particularly difficult endeavor that becomes even more so, as one crosses the great evolutionary divide between vertebrate and invertebrate phyla. Here, we explore the possibility of consciousness in one group of invertebrates: cephalopod molluscs. We believe such a review is timely, particularly considering cephalopods' impressive learning and memory abilities, rich behavioral repertoire, and the relative complexity of their nervous systems and sensory capabilities. Indeed, in some cephalopods, these abilities are so sophisticated that they are comparable to those of some higher vertebrates. Following the criteria and framework outlined for the identification of hallmarks of consciousness in non-mammalian species, here we propose that cephalopods - particularly the octopus - provide a unique test case among invertebrates for examining the properties and conditions that, at the very least, afford a basal faculty of consciousness. These include, among others: (i) discriminatory and anticipatory behaviors indicating a strong link between perception and memory recall; (ii) the presence of neural substrates representing functional analogs of thalamus and cortex; (iii) the neurophysiological dynamics resembling the functional signatures of conscious states in mammals. We highlight the current lack of evidence as well as potentially informative areas that warrant further investigation to support the view expressed here. Finally, we identify future research directions for the study of consciousness in these tantalizing animals.

## Introduction

The notion that an animal like the cephalopod mollusc *Octopus vulgaris*, an invertebrate, and its allied species (e.g., cuttlefish and squid) could have anything remotely resembling subjective experience is quite likely to be met with astonishment in some quarters. The suggestion that these animals might have a sophisticated form of consciousness would, to many, be shocking. However, from a purely theoretical perspective, the subjective experiences which we, as humans, frequently report can only lead us to *presume* that other humans have consciousness, just as we *presume* the existence of features of the external world (Humphrey, 2006; Andrews, 2020). By this line of thinking, there is no reason to deny that many other animals experience at least some degree of primary consciousness, contingent, of course, on their sensory, cognitive, physical, and life faculties and constraints (e.g., Feinberg and Mallatt, 2020).

Consciousness has long been imagined to be very similar to what has been termed a ‘first principle' in mathematics: a concept intrinsic to everyone which cannot be explained by formal logical systems because our conventional language is incapable of encompassing or expressing it. It is a concept that seems to vacillate between the domains of philosophy, science, and, sometimes, human morality (Vitti, 2010; Seager, 2016). Notably, William James was the first to objectively define consciousness not as a concept or thing, but rather as a process resulting from the complex interaction of brain, body, and environment (James, 1977). This definition helped to establish a dynamic vision of consciousness as the subjective experience of an individual that does not necessarily require explicit terms to be recognized, but rather relies on a specific, relatively complex neural organization, potentially extending this faculty to those non-human vertebrates possessing similar - or homologous - organization. Undoubtedly, having established that consciousness is a process that is endemic to the biological world, the next step might be to analyze it using Tinbergen's four questions in an effort to define its adaptive, phylogenetic, causal, and ontogenetic properties (Gutfreund, 2018).

The neuroscientist Gerald Edelman offered a compelling vision of consciousness as a process contingent on richly interconnected and reentrant neural circuits capable of integrating an extraordinary number of inputs from both external environments and internal milieus. In this view, consciousness arises through the emergence of widespread, temporally linked mappings of multimodal extrinsic and intrinsic signals, i.e., sensory binding. According to Edelman, it is likely that natural selection shaped the structures and systems that produced consciousness (e.g., thalamocortical and cortico-cortical circuitry and the limbic system, among others) through a continuous fine-tuning process over millions of years (Edelman, 2003). This becomes even clearer when we consider the adaptive value of neural systems: not a merely a set of instructions, but rather, highly selective pathways of widely distributed populations of neurons - or neuronal groups - with the ability to integrate as much information as possible and process it very quickly (see Neuronal Group Selection; e.g., Neural Darwinism, in: Edelman, 1987, 1989, 2003).

Though the vision of consciousness as one consequence of rewiring and neuronal plasticity may be hard to accept for those who have claimed consciousness as the quality that separates humans from the rest of the animals, it will be even more challenging to determine if there is enough evidence accumulated in the last decade to suggest that it is not a uniquely human faculty or even an exclusive property of vertebrates. One empirical approach to the study of consciousness is to consider behavioral abilities as indicators of consciousness; despite the absence of verbal language, there are specific solutions for investigating consciousness in non-human animals (as well as human neonates) and, in particular, demonstrating the requisite degree of neural complexity, plasticity, and behavioral flexibility for subjective experience in non-human mammals, birds, some reptiles, and even certain invertebrates (Edelman et al., 2005; Seth et al., 2005; Edelman and Seth, 2009; Vitti, 2010).

Here, we review several theories of and proxies for consciousness with a particular focus on cephalopods molluscs. We highlight the strengths, drawbacks, and lacunae when considering these animals as candidates for a distinct level or degree of consciousness. The topic has been covered previously in a series of papers (Mather, 2008, 2021a,b; Edelman and Seth, 2009; Birch et al., 2020; Feinberg and Mallatt, 2020). We will highlight the essential aspects of these works.

## Cephalopod Consciousness: A Short Overview of Others' Contributions

Cephalopods regularly challenge our assumptions about the limits of invertebrate cognition and behavior, underline the borders of our current knowledge base, and surprise us with their unique and rich repertoire of capabilities that in some instances equal or even exceed those of certain vertebrates. Sophisticated visual and tactile learning (for review see e.g., Sanders, 1975; Marini et al., 2017), as well as a kind of spatiotemporal awareness, may indirectly support the idea that cephalopods possess a form of sensory consciousness (as suggested by Mather, 2008). Perhaps the most intriguing aspect is their almost complete lack of stereotyped behaviors or fixed action patterns. Indeed, the development of well-oriented flexible responses to changes in stimuli or environmental contexts to mention some (e.g., Sanders and Young, 1940; Maldonado, 1963b, 1965; Messenger, 1973; Sanders, 1975; Darmaillacq et al., 2004; Agin et al., 2006; Marini et al., 2017; Hanlon and Messenger, 2018) suggests that cephalopods employ domain specificity - as recently proposed by Birch et al. (2020) and overviewed by Mather (2021a,b) -, a faculty strongly associated with active brain processing (Hirschfeld and Gelman, 1994) and, as we will further mention in this paper, a theory of mind (ToM), i.e., the ability to intuit the thoughts and beliefs of others by a sort of ‘mind reading' faculty that requires some neural encoding of social domains (e.g., Frith and Frith, 2006; Apperly, 2011; for cephalopods see Godfrey-Smith, 2013).

As reviewed by Edelman and Seth (2009) - and particularly at the behavioral level - consciousness has been associated with behavioral flexibility (e.g., plasticity), among other attributes. Marked learning capabilities (review in e.g., Sanders, 1975; Marini et al., 2017; Hanlon and Messenger, 2018; Gutnick et al., 2021) and interindividual differences in temperament have been observed across some cephalopod species (Mather and Anderson, 1993; Sinn et al., 2001, 2008, 2010; Sih et al., 2004a,b; Adamo et al., 2006; Scheel et al., 2017; Zoratto et al., 2018; Borrelli et al., 2020; O'Brien et al., 2021). It is noteworthy that the existence of ‘personalities' in octopus and other cephalopods has been considered an ´interesting manifestation of individual differencesˇ, but questioned as a demonstration of ´consciousness or self-monitoringˇ (Mather, 2008, p. 43). However, such a spectrum of temperaments termed the ‘shy-bold continuum' (Wilson et al., 1994; for cephalopods see for example: Borrelli and Fiorito, 2008; Borrelli et al., 2020), suggests that each individual develops its own unique response to a given stimulus, forming a specific workspace and an appropriate representation of the environment (Baars, 1994; Edelman et al., 2005).

Although the large body of evidence for high-level behavioral abilities might suggest the representation of a multimodal set of perceptual and motor events in the cephalopod nervous system, this alone would not be sufficient to make the case for primary consciousness. For one thing, reported behavioral indicators may occasionally be misinterpretations - often due to the application of anthropomorphism - of what was actually observed, and as such, could skew our perspective (Gutfreund, 2019). For another, advanced cognition doesn't necessarily coincide with conscious experience, even in human beings (Vallortigara, 2017). Given these caveats, what other reliable indices or proxies can we employ to probe for consciousness in cephalopods?

It has been persuasively argued that in order for animals to evince any degree of consciousness, they must possess highly elaborated neural structures capable of generating a ‘global workspace' in which sub-networks of signals (or percepts) from otherwise disparate inputs are bound together within a single dynamic network. This vast network, comprising a conscious gestalt of bound percepts, may then be broadcast widely throughout the brain, at which point complex responses can be elicited (Baars, 1994). The Global Workspace Theory (GWT) was clearly formulated with the mammalian brain in mind. GWT proposes that conscious gestalts arise specifically from signaling both within the cerebral cortex and between cortex and thalamus. The theory has been further refined through physiological and imaging data gathered primarily from human subjects (Baars, 1994, 2002; DeHaene and Changeux, 2004; Dehaene and Changeux, 2005).

Given the foregoing, is it reasonable to extend GWT to investigations of consciousness in animals quite distant from the mammalian (or even vertebrate) line?

Seth et al. (2005) proposed 14 criteria (actually 17: three well-established brain correlates + 14 distinctive properties; see also Table 1) for consciousness in humans and non-human mammals, including specific neuroanatomical features and physiological markers, that - with proper considerations and the right tools - could be objectively applied to non-mammalian phyla as well (Edelman et al., 2005). Extending this framework to cephalopods, a logical starting point would be the identification of reliable objective neural correlates analogous to those observed in conscious, awake vertebrates.

**Table 1:** Key features to access consciousness dimensions, and dimensions and hallmarks of consciousness (modified after: Edelman et al., 2005; Seth et al., 2005; Edelman and Seth, 2009; Birch et al., 2020).

**Key features for assessing dimensions of consciousness**		
	**Features**	**Notes**
	EEG signatures	Evidence in cephalopods: Fast, irregular electrical brain activity; compound field potentials, and evoked potentials (Bullock, 1984; Bullock and Budelmann, 1991; Brown et al., 2006; see also Butler-Struben et al., 2018)
	Cortex and thalamus	Evidence in cephalopods: existence of functional analogs identified at the level of the superior frontal-vertical lobe systems and dorsal basal lobe (supra-esophageal mass; for review see Shigeno et al., 2018)
	Widespread brain activity	For cephalopods see: Bullock (1984); Bullock and Budelmann (1991); Brown et al. (2006); Butler-Struben et al. (2018)
**Wide Range**		
**Dimensions**	**Hallmarks**	**Notes**
p-richness	Sensory binding	Ability to perceive different features of the environment (e.g., shape, taste, odor). For review in relationship to cephalopods, see Birch et al. (2020), and Mather (2021b); see also e.g., Chiao and Hanlon (2001a,b), Scatà et al. (2017), Mezrai et al. (2019), van Giesen et al. (2020).
	Facilitation of learning	Conscious perception and learning of temporal relationships. As reviewed by Birch et al. (2020), and also Mather (2021b) for cephalopods; see also, e.g., Marini et al. (2017); Borrelli et al. (2020), Bublitz et al. (2017) Schnell et al. (2021a). In regards to neural correlates of sensory and learning processing/capacities in cephalopods, note the large number of studies based on the impairment of the neural circuitry underlying visual and chemo-tactile memory-systems (for review see: Sanders, 1975; Borrelli and Fiorito, 2008; Marini et al., 2017).
e-richness	Accurate reportability	For review in relation to cephalopods, see Birch et al. (2020). Conscious contents are reportable by several behavioral responses following evaluation. Such valence should be applied in affectively based decision making.
	Informativeness	Some animals may continually evaluate small changes in their internal milieus and external environments, while others may only react to substantial changes and ignore redundant stimuli. For review, see also: Marini et al. (2017), Hanlon and Messenger (2018)
	Focus-fringe structure	“Fringe Conscious” (e.g., Norman, 2017) events, like feelings of familiarity and experiences having emotional valence (e.g., the “tip of the tongue phenomenon”) are consistent with the idea that the self is an interpreter of conscious experience rather than a primary source of perceptual content.
Unity	Subjectivity	For review in relation to cephalopods, see Birch et al. (2020) and also Mather (2021a). Consciousness is marked by the existence of a private flow of events accessible only to the experiencing subject, despite being reportable. The world and all the experiences generated by our brain have a common subject.
	Internal consistency	Consciousness is marked by a consistency constraint. That is, even when similar stimuli are presented simultaneously, only one can become conscious at any given time (in relation to our “inner” common subject).
	Limited capacity and seriality	Consciousness flows from one scene to another in a serial manner and is constrained to just one scene at any given moment.
	Self-attribution	Consciousness is experienced by an observing self.
		As reviewed by Birch et al. (2020), behavioral and neural asymmetries have been reported in cephalopods to different extents (Jozet-Alves et al., 2012a; Schnell et al., 2016a; Frasnelli et al., 2019). Cephalopod molluscs are capable of parallel processing of visual and/or tactile inputs (e.g., Borrelli, 2007; Schnell et al., 2018; Borrelli et al., 2020), possibly including recognition (e.g., Tricarico et al., 2011, 2014; Nesher et al., 2014; Katz et al., 2021), but also in the process of integrating information from both eyes (e.g., Feord et al., 2020; see also El Nagar et al., 2021).
Temporality	The rapidly adaptive and fleeting nature of conscious scenes	Birch et al. (2020) mention some cephalopod studies that may address this dimension (e.g., Billard et al., 2020a,b; Poncet et al., 2020; Schnell et al., 2021a,b). Immediate experience of sensory past and cognitive present that persists for a few seconds, forming a continuous stream of events.
	Stability of contents	Conscious contents are stable, even though experiences can be temporally integrated across longer timescales—comprising past and future events—in what it might be termed “temporal dimensions.”
Selfhood	Conscious knowing and decision making	Consider here: Crook and Walters (2014); Birch et al. (2020); and for example (Tricarico et al., 2011, 2014; Nesher et al., 2014). Consciousness is useful for generating knowledge about the world around us, as well as that of our own internal states and processes, all of which can influence decision making.
	Allocentricity	The foregoing implies the discrimination of ourselves from the external world by an allocentric faculty which makes use of neural representations of external objects to build conscious scenes.

*Wide range: Consciousness has an extraordinary range of different contents, including perception in the various senses, endogenous imagery, feeling states, inner speech, concepts, action-related ideas, and “fringe” experiences such as feelings of familiarity. Examples of studies testing a given feature, - suggesting the possibility of a given hallmark in cephalopods, within a specific dimension are based on the recent review by Birch et al. (2020) and our own coverage of the scientific literature*.

At the level of gross morphology, invertebrates such as cephalopods lack neural structures resembling cortex or thalamus, but this does not preclude the existence of structures that carry out functions closely analogous to those of cortex and thalamus (Shigeno et al., 2018).

Indeed, the architectural complexity of certain neural structures in cephalopods approaches that of higher vertebrates, and the specific organization of those structures suggests they may be functional analogs of mammalian brain areas implicated in the instantiation of conscious states. In particular, the dorsal basal and subvertical lobes receive disparate inputs from throughout the body *via* both direct and indirect pathways from the suboesophageal mass, which acts as a relay center - akin to the thalamus—conveying signals to the frontal and vertical lobes. Intriguingly, the purpose of these lobes is to integrate such inputs and produce an elaborate response: a function similar to that performed by the mammalian cortex (see Shigeno et al., 2018 and below).

According to Carls-Diamante, despite the outstanding cognitive abilities that might underlie a sort of ‘mentality,' cephalopod consciousness may not be organized as a ‘united field' (Carls-Diamante, 2017, 2021), but rather as a “community of minds” (Schwartz, 2019). The assumption of Carls-Diamante derives from the so called ‘isomorphism thesis,' which relates the structure of an animal's consciousness to the structure of its nervous system (Bayne, 2010).

The atypical neuroanatomy of cephalopods, composed of a central brain and a complex peripheral nervous system - comprising about two thirds of the total number of neural cells (in the octopus; e.g., Young, 1963; Graziadei, 1971) supports the view of an embodied organization of the octopus nervous system (Hochner, 2013). Such independence from the brain mass (i.e., the neuro-motor system in the arms; Sumbre et al., 2001, 2005, 2006) could arguably be sufficient to rule out the possibility that cephalopods integrate different experiences into a perceptual unity (Carls-Diamante, 2017). However, the structure of subjective experience conceived as a united conscious field is too simplistic, and it can hardly be generalized to the organization of many species' sensorimotor systems, as these are very different from one another (van Woerkum, 2020). Thus, with these assumptions in mind, what cephalopod experience can only be understood by investigating how their body, nervous system, and sensory equipment allow them to actively and flexibly interact with - and, integrate inputs from - the environment (Godfrey-Smith, 2019; van Woerkum, 2020; see also Masciari and Carruthers, 2021).

In a recent attempt to address the issue of consciousness in non-mammal animals, Birch et al. (2020) suggested a framework comprising five dimensions that draws from examples provided by the octopus and other closely allied invertebrate candidates. In this synthesis, the five dimensions are: perceptual richness (p-richness), evaluative richness (e-richness), integration both at a given time (unity) and across time (temporality), and self-awareness (e.g., selfhood). Notably, a discussion around p-richness in octopus has recently been initiated by Mather (2021b). Table 1 lays out a possible correspondences between these five dimensions and the criteria specified by Seth et al. (2005).

In what follows, we will further explore these issues and argue that if subjective experience requires some minima of network organization and computational power and primary consciousness can be imputed from simple sensory and cognitive representations, then a cephalopod like the octopus - with its 500 million neurons - large and highly differentiated central nervous system, and rich behavioral repertoire and flexibility, must be considered as a fruitful model organism for investigating consciousness beyond the vertebrate lineage. And so begins our journey to the biological frontiers of awareness on the backs of the cephalopod molluscs.

## Cephalopod Cognition

### Prolog: A Narrative Arc From Aristotle and Darwin to the Present

Throughout the history of natural observation and exploration, cuttlefish, squid, and octopuses (the most thoroughly studied of all cephalopods) have provided ample and compelling evidence that they are more than simply eight-armed (plus two tentacles in the case of cuttlefish and squid) masses of muscles guided by a primal insatiable appetites.

For many observers through the ages, these animals have offered ample cause for surprise, exhibiting behaviors that ultimately contributed to their steady rise in public awareness over the past century (Lee, 1875; Lane, 1960; Cousteau and Diolé, 1973; Mather et al., 2010; Courage, 2013; Schweid, 2013; Montgomery, 2015).

Apart from Aristotle - who in the fourth century BC expressed the opinion that the octopus was a curious, but stupid animal (Aristotle, 1910; overview in Marini et al., 2017) - recorded observations of, and anecdotes about cephalopods and their astonishing capabilities are ubiquitous throughout recorded history (for review see for example, Borrelli and Fiorito, 2008; De Sio, 2011; Dröscher, 2016; Marini et al., 2017). Interestingly in *The Voyage of the Beagle*, Darwin reported an encounter with an octopus on the coast of Cape Verde. He noted that the octopus was not only able to withstand his gaze, but also seemed to stare back at him intently in a kind of match of attentional wits (Darwin, 1870).

Octopuses were depicted in ancient bestiaries as voracious, cunning, and positively evil (see for example: Lee, 1875; Chapko et al., 1962), based on the broad attribution of moral categories to animals. Setting aside this compellingly colorful characterization, a considerable number of octopus tales, including Darwin's, have, over the centuries, contributed to cementing the reputation of the octopus in Western culture (Lane, 1960; Caillois, 1973; Hochner et al., 2006; Borrelli and Fiorito, 2008; Makalic, 2010; Hochner, 2012; Marini et al., 2017).

In the first century AD, Pliny the Elder (Pliny, 1961) reported witnessing an octopus waiting patiently for a large shellfish (*Pinna nobilis*) to open its valves in order to prop it open with a stone it held in its arms. The veracity of Pliny's observation was corroborated centuries later by the personal account of the zoologist Jeannette Powers (Power, 1857; for an overview see: Borrelli and Fiorito, 2008; Marini et al., 2017).

With the advent of marine stations and inland aquaria, cephalopods became more readily amenable to observation and experimentation, and a wealth of comparable histories emerged, based on more or less systematic and increasingly frequent observations (De Sio et al., 2020). For example, there are anecdotal accounts of cephalopods that recognized individual conspecifics, performing body patterns to communicate, exhibited individual temperaments, and even recognized individual humans (Romanes, 1885) possibly forming a kind of affective bond.

Following from such compelling anecdotal accounts, experimental trials have provided proof of such sophisticated capabilities in cephalopods. Among the most notable findings are: the recognition of human faces (Anderson et al., 2010) and individuals (Boal, 2006; Tricarico et al., 2011, 2014), play (Mather and Anderson, 1999; Kuba et al., 2003, 2006), ‘personality' (Mather and Anderson, 1993; Borrelli, 2007; Borrelli and Fiorito, 2008; Sinn et al., 2008, 2010; Borrelli et al., 2020; O'Brien et al., 2021), social learning (Fiorito and Scotto, 1992; Fiorito, 1993; Fiorito and Chichery, 1995; Amodio and Fiorito, 2013; Huang and Chiao, 2013; Tomita and Aoki, 2014), episodic memory (e.g., Pronk et al., 2010; Jozet-Alves et al., 2013; Schnell et al., 2021b), and deliberate and projective tool use within a specific octopus population (Finn et al., 2009), among others.

### Discriminatory and Anticipatory Behaviors

A strong link between perception and memory, together with the functional neural circuitry underlying such a link, have been proposed as necessary requisites for conscious processing (Edelman, 1993; Edelman et al., 2011). Since discriminatory and anticipatory behaviors suggest such a link, we will now turn our focus to evidence for these behaviors in cephalopods.

As reviewed extensively by Marini et al. (2017), the breadth of learning paradigms in which cephalopods have demonstrated their cognitive capabilities is quite remarkable (see Table 3 in Marini et al., 2017; for review see also Hanlon and Messenger, 2018). Indeed, the vast majority of the learning studies carried out on cephalopods have relied on their well-characterized predatory behavior. Such behavior has been leveraged both to study the recovery of predatory performance following capture and to evaluate the possible interference of various stimuli or contexts with the animals' attack response. As a consequence, an established practice of learning paradigms for octopuses and other cephalopods (mainly *Sepia officinalis*) is that given experimental protocol should start after a period of ‘acclimatization' (sensu Boycott, 1954; Maldonado, 1963b,c) for an animal in a captive situation. It is since the pioneering studies initiated at the end of the 1940's up to recent times, the acclimatization period is a variable length of time during which the animal is exposed to a novel environment (e.g., the tank and its surroundings) and presented with a live prey or conditioned to attack dead prey (Amodio et al., 2014; Fiorito et al., 2015). The ‘acclimatization' (i.e., acclimation) is considered a form of contextual learning (Maldonado, 1963a,b,c, 1965; Borrelli, 2007; Marini et al., 2017; Borrelli et al., 2020). Trials with cuttlefish, octopus, and in some cases squid, have shown that the daily presentation of food increases the likelihood that the animal will attack. Predatory performance, measured as the time to attack prey from its appearance in the tank, thus improves over time. This phenomenon reveals an important feature of both ‘positive' and ‘negative' learning capabilities (Maldonado, 1963b, 1965). Notably, this process is regulated mainly by the vertical lobe system, as shown in octopus (Maldonado, 1963a; review in Sanders, 1975). Also evident from these studies are the differences between individuals; as contextual learning progresses, other characteristics of the subjects may become apparent, with inter-individual differences emerging in response (e.g., readiness) to stimuli (Borrelli, 2007; Borrelli et al., 2020). Of course, it should be noted that differences between species are to be expected, owing to different lifestyles and adaptive capabilities (Nixon and Young, 2003; Hanlon and Messenger, 2018; Ponte et al., 2021).

Moreover, the exposure to a novel laboratory environment - e.g., the tank and/or experimental setting - involves the confinement of a given animal to a space that comprises a much smaller foraging area than that encountered in the wild. The animal is thus immersed in a comparatively monotonous captive setting, regardless of the degree of enrichment provided. Under these circumstances, evidence of the extreme breadth of cephalopod behavioral plasticity again comes to the fore. Frequently, animals have been presented with tasks - even those spaced across different trials - designed to assess their predatory behavior (e.g., attack/non-attack or take/reject responses). In such cases, they have generally adapted their species-specific predatory response to the new context. This type of contextual learning takes a variable amount of time and depends on the species being investigated, the animals' previous experiences, individual variability due to ecological and biological factors, including developmental and life cycle stages (e.g., differences in age, sex, maturity, etc.), neophobia, interindividual variability in behavioral responses (e.g., temperament), and plasticity (Borrelli et al., 2020), among others. Such studies of predatory behavior once again provide clear examples of a positive learning process (Maldonado, 1963b, 1965).

Individual and social learning have been widely explored in cephalopods (Sanders, 1975; Marini et al., 2017; Hanlon and Messenger, 2018) and, as previously noted, in all cases animals have exhibited a high degrees of plasticity and adaptability in their behavior. A few examples bear mentioning here.

Addition of quinine (a bitter taste substance) to the carapace of presented prey, such as crab or shrimp, resulted in rapid learning of taste aversion in *Sepia officinalis*; this facilitated the animals' future choice of prey and behavioral responses were retained over long durations (Darmaillacq et al., 2004). Images of a potential predator (e.g., a bird) gliding over the tank elicited startle reactions in cuttlefish (Calvé, 2005), which also affected future hunting behavior (Adamo et al., 2006). Successive visual discrimination tasks have generally been used as training protocols for octopus (for review see for example: Sanders, 1975) as well as cuttlefish in which autoshaping has been demonstrated (Cole and Adamo, 2005). The foregoing provide further examples of the classic training paradigm that has been well-established for cuttlefish (i.e., the “prawn-in-the-tube;” e.g., Sanders and Young, 1940; Messenger, 1973; Agin et al., 2006; Purdy et al., 2006; Cartron et al., 2013) and the visual (and tactile) discrimination tasks or problem-solving paradigms that have been developed for octopuses over many years (Sanders, 1975; Wells, 1978; Marini et al., 2017).

Memory retrieval is the fundamental basis for an individual's ability to benefit from past experiences. In some cases, though, it proves particularly useful in referencing a specific episode and where and when that episode occurred. In addition to studies in birds and mammals, recent work in cuttlefish has shown that these animals remember what they ate, as well as where and how long ago they ate, thus satisfying the “what,” “where,” and “when” criteria for episodic-like memory (Crystal, 2010; Jozet-Alves et al., 2013; see also e.g., Schnell et al., 2021b). Furthermore, while episodic memory refers, in a sense, to the ability to time-'travel' to an individual's past, retrieving specific features belonging to such memories is a cognitive capacity that involves the contextual activation of source-memory processes. In other words, there must necessarily be semantic processes in play that afford the retrieval of a memory and its origin, as well as an indexical comparison with other stored information in order to distinguish different episodic memories from one another. Studies of *S. officinalis* proved these animals' ability to discriminate between visual and olfactory modalities and then recall which one was previously encountered before an extended delay (Billard et al., 2020a).

Notably, John Zachary Young (JZ) failed to establish definitive evidence in support of the integration of sensory modalities in octopus during learning (e.g., visual vs chemo-tactile; Young, 1991, 1995), despite extensive overlap in the neural circuitry mediating the two sensory-motor learning and memory systems, as well as some behavioral indications (Marini et al., 2017). In this regard, it would be useful to refer to the matrix-like functional organization of cephalopod nervous systems. Multiple matrices occur in regions of the central nervous systems of cephalopods. These control behavioral responses, allowing signals of different types (e.g., visual, chemo-tactile) to interact to some degree and regulate subsequent behavior - in particular, the attack/take and retreat/reject responses (Young, 1961, 1964; Maldonado, 1963c; Packard, 1963). These systems of matrices work by modulating promotion and/or inhibition of specific responses. Overall, they are tuned to facilitate the exploratory behavior that characterizes these animals. According to Young (and as emphasized on many occasions in this review), cephalopod matrix systems bear more than a passing resemblance to regions of the mammalian nervous system, in particular the limbic lobe and neocortex (Young, 1995). Despite some indications of more extensive comingling of signals, Young concluded that complete integration - or transfer - between the visual and tactile information matrices occurred only at the effector level. However, in preliminary experiments, Allen et al. (1986) showed that limited cross-modality does indeed occur (but see also Anderson and Mather, 2010). Given the limited nature of recent evidence from cuttlefish and the need for further studies of octopus sensory integration, we can only conclude that cross-modality is a long-standing issue in cephalopod cognition which warrants further systematic study. Nevertheless, the degree of behavioral complexity shown by cephalopods provides a strong rationale for exploring the possibility of conscious experience in these invertebrates.

Cephalopods commonly adopt dynamic, flexible predatory strategies that include selective, opportunistic, and plastic foraging behaviors in response to changing environmental conditions (Hanlon and Messenger, 2018). Apropos of such flexibility, recent lines of research have addressed whether cephalopods exhibit future-oriented behaviors or are capable of planning. By definition, future-oriented planning in animals (e.g., Clayton et al., 2003) requires behavior to be flexible and dependent on, or sensitive to, consequences. In cuttlefish, Billard et al. (2020b) have shown that animals adapt their behavior to environmental conditions on a daily basis. Moreover, a certain food choice in one moment of the day can determine the dietary choice in a following moment, increasing variation (an instance of food devaluation). To rule out the possibility that an animal's future planning depends on relative or contingent motivational states, other experiments have been carried out, showing that preference of *S. officinalis* for a shrimp in a quantity comparison test occurs *via* learned evaluation that depends on the relative value of previous prey choices (Kuo and Chiao, 2020). In addition, self-control and tolerance of delays in receiving a reward - which are well-characterized features of mammals with elaborate inhibitory neural circuitry - are also documented in this species (Schnell et al., 2021a). Finally, we can expect other paradigms (e.g., maze learning) and model organisms (e.g., octopus) to shed further light on cephalopods' capacity for future-oriented behaviors and planning (Poncet et al., 2020).

The foregoing behavioral paradigms and tests of cephalopod capabilities are largely based on their predatory response, an aptitude which relies mainly on visual cues, as well as chemo-tactile information (Yarnall, 1969; for review see: Sanders, 1975; Borrelli and Fiorito, 2008; Marini et al., 2017; Villanueva et al., 2017; Hanlon and Messenger, 2018; see also Maselli et al., 2020).

As visibility in water may often be limited, chemical cues can provide alternative reliable signals that aquatic animals can use, even for the identification of conspecifics. This is the case for cephalopods (Huffard and Bartick, 2015; Polese et al., 2015; Morse et al., 2017; Morse and Huffard, 2019) as well as a wide variety of other invertebrate and vertebrate phyla, including crustaceans, insects, and fish (Hepper, 1986; Cannicci et al., 2002; Gherardi et al., 2010, 2012; Sheehan and Tibbetts, 2011). Moreover, the sense of touch, which may be linked to taste, plays an important role in octopus foraging and learning (Chase and Wells, 1986; Mather and O'Dor, 1991; Forsythe and Hanlon, 1997; Godfrey-Smith and Lawrence, 2012; van Giesen et al., 2020) and is also involved in some social interactions (Huffard et al., 2008; Amodio and Fiorito, 2013; Caldwell et al., 2015; Huffard and Bartick, 2015; Scheel et al., 2016; Morse et al., 2017).

For example, Tricarico et al. (2011) found that octopuses performed a higher number of physical contacts when placed in an arena with conspecifics they had never encountered before the testing condition, in contrast to those that had previous experience with the ‘dear enemy' on the other side of a transparent barrier during a preliminary acclimation phase of the experiment (Tricarico et al., 2011).

As highly developed as cephalopods chemo-tactile faculties may be, it is their visual faculties that stand out among the invertebrates, rivaling even those of some higher vertebrates (e.g., Packard, 1972; Hanlon and Messenger, 2018). Complex vision allows animals to negotiate a wide variety of ecological and biological challenges, including predation, navigation, discrimination learning, some forms of proprioception (Wells, 1960; Gutnick et al., 2011), and even intraspecific communication. A graded diversity of color, texture and postural components forms the basis for the body patterns emitted over longer or shorter periods of time (Packard and Sanders, 1971; Packard and Hochberg, 1977; review in: Borrelli et al., 2006; Hanlon and Messenger, 2018). While body patterning allows effective mimicry and disguise, among other functions, it also provides a channel for intraspecific communication (Hanlon et al., 1999; Shashar et al., 2004; Schnell et al., 2016b), including hidden or ‘secret' signals to other species (e.g., Mäthger and Hanlon, 2006; review in e.g., Tricarico et al., 2014; Hanlon and Messenger, 2018).

In many species, including a wide variety of primates and birds, vision is the primary sense used to distinguish individuals by specific facial attributes, recognize emotions by body posture and facial expression, and control gaze direction; a faculty often exploited by researchers in investigations of ToM in non-human animals (Bugnyar et al., 2004; Carter et al., 2008; Wilkinson et al., 2010; Grossmann, 2017; Nawroth et al., 2017; Kano et al., 2018). Indeed, a seemingly complex test termed “reading the mind in the eyes” has been devised to study how an adult human is able to assign a complex mental state to another simply by looking into his eyes (Baron-Cohen et al., 2001). A cursory survey of the comparative literature (see above) suggests that this aspect of ‘mind-reading' may have its earliest antecedent in the sensitivity of gaze direction.

Certainly, the use of vision to recognize individuals has an ancient origin. Notably, insects such as the social wasp *Polistes fuscatus* (Tibbetts, 2002) and crustaceans such as lobsters (Gherardi et al., 2010), crayfish (Van der Velden et al., 2008) and crabs (Cannicci et al., 2002) are able to identify individual conspecifics based on unique visual facial cues.

Despite our limited understanding of social (and individual) recognition in octopuses and other cephalopods (Boal, 2006; Tricarico et al., 2011, 2014), accumulating evidence suggests the existence of a complex vision-based modality that mediates interactions between individuals (see above; Packard and Sanders, 1971; Kayes, 1974; Tricarico et al., 2011; Scheel et al., 2016; Schnell et al., 2016b). Neighbors typically show few agonistic interactions with each other, suggesting that they are affected by the “dear enemy phenomenon,” i.e., a reduced aggressiveness toward neighbors in territorial animals (*sensu* Fisher, 1954), a phenomenon that has also been observed in birds, mammals, and many other vertebrates, as well as in a number of invertebrates (Tibbetts and Dale, 2007; Snijders and Naguib, 2017). The finding by Tricarico et al. (2011) that *O. vulgaris* can recognize conspecifics, discriminate known from unknown individuals, and remember the discrimination, indicates that this species is capable of at least class or binary individual recognition (Tibbetts and Dale, 2007), an ability not yet demonstrated in other cephalopod species. The ability to recognize and remember ‘opponents' and conspecifics may be of adaptive value to octopuses, as it is likely the proximate mechanism regulating the ‘dear enemy' phenomenon. This may explain the rare interactions between octopuses observed in the field (Tricarico et al., 2011). Despite the need for more in-depth studies to determine whether these animals are capable of true individual recognition, the work of Tricarico et al. provides to the best of our knowledge, the only known account of conspecific social recognition for this taxon (Tricarico et al., 2011, 2014). This study is comparable to the brief report by Anderson et al. (2010) that octopuses are capable of recognizing individual caretakers in the laboratory, confirming the ancient anecdote about cephalopods mentioned earlier (Schneider, 1880; Romanes, 1885).

Some animal species have been reported to be able to differentiate among humans by their faces; an ability not limited to domesticated species (Boivin et al., 1997; Tanida and Nagano, 1998; Rybarczyk et al., 2001; Racca et al., 2010; Stone, 2010; Nagasawa et al., 2011; Müller et al., 2015; Wood and Wood, 2015), but also observed in invertebrates such as the honeybee and other insects (Dyer et al., 2005; Avarguès-Weber et al., 2017). If recognition of individual humans is confirmed in the octopus, this could provide further evidence of the cognitive distinctiveness of these animals among invertebrates.

## Young's Cephalopod Model of the Brain and the Search for Consciousness in Cephalopods

Young (1954) famously proposed the octopus brain as a useful general model for the study of learning and memory, as it was considered both a tractable object for experimental study (e.g., a nervous system much simpler than our own) and a pointedly epistemic - even rhetorical - device that enjoins the researcher to find novel ways to discuss physical phenomena. In his view, these ways departed from our tendency as humans to frame everything in psychological terms when speaking about ourselves. In this sense, JZ claimed to be following the example of Ryle (1949). Of course, there is a vast semantic and epistemological chasm between the search for memory in a mollusc (even a cephalopod) and assessment of its higher cognitive faculties, from mind-reading to consciousness. First of all, the very concept of ‘memory' needed to be progressively re(de)fined by Young to afford comparisons across the animal kingdom, as well as with genetically and electronically based memory systems (see De Sio, 2011 and cited works therein). Understandably, such a perspective might surprise the contemporary reader. We are accustomed to speaking - almost reflexively so - of the memory of a computer, the memory of our immune system, or the memory stored in our genes, often without acknowledging the analogical heavy lifting involved in drawing this seemingly simplistic equivalence. In the search for memory and its engram, a link with mechanical causality was attempted for analytical purposes by Young using octopus as the biological platform (Young, 1951).

Young considered the octopus as the animal possessing a brain appearing the ´most divergent from that of mammals that is really suitable for study of the learning processˇ (Young, 1971, p. vii). The phenomenological proximity of behavioral traits to, and vast phylogenetic distance from, vertebrates convinced Young and his many collaborators to consider cephalopods, especially *O. vulgaris*, to be suitable general model of the brain (Young, 1964). As reviewed in Marini et al. (2017), Young and Boycott began their explorations of *O. vulgaris* at the Stazione Zoologica (Naples, Italy) in the spring of 1947, starting from scratch and based on scarce and largely unsystematic precedents. Their aim was to study learning in these animals by combining behavioral observations and surgical ablation of selected parts of the neural centers, in order to explore and define the higher functional organization of the octopus' brain and its control of behavioral outcomes. After many years of intensive study and systematic experimentation, Boycott presented an efficient - and simplified - training technique in which all the possible outcomes (e.g., complexity, individuality, and ambiguity of behavioral responses) were not considered. The predatory response of *O. vulgaris* (as in other cephalopods, e.g., Sanders and Young, 1940; Messenger, 1973) was exploited as a bio-behavioral key for teaching animals to discriminate between positively and/or negatively reinforced stimuli; in other words, to make choices and decisions in a given situation. Tens of trials were sufficient for the animals to learn the task and respond correctly in a fairly stable and predictable manner (review in Marini et al., 2017). Training animals to discriminate between different shapes by simultaneous and/or successive presentation of two discriminanda, proved successful (Sanders, 1975). The original training protocol was refined several times until it was finally standardized, such that octopuses: (i) are given a period of acclimation in the tanks; (ii) after acclimation, wait in their den until a stimulus enters their tank and elicits a response; and (iii) after a short period of attention, that response triggers either ‘retreat' or ‘attack' (Maldonado, 1963b,c, 1965; Packard, 1963). Of course, ‘attack' and ‘retreat' are not the only elements of the story; decreasing time needed to respond to the stimulus, ‘attention', ‘cautiousness', ‘incomplete-attacks', ‘shyness', and ‘boldness' represent the complex behavioral responses exhibited by an octopus during training (Maldonado, 1963b, 1965; Borrelli, 2007; Borrelli et al., 2020). The versatility of the octopus training paradigms fostered the growth of the field well into the 1960's, with several directions of research evolving from the original work (see Figure 1 in Marini et al., 2017). These studies demonstrated that the octopus was capable of a diversity of learning capabilities (Sanders, 1975; Marini et al., 2017). Through this work, Young's goal was achieved: he was able to build a detailed model of the brain of a learning and behaving octopus (Young, 1961, 1964). Young was inspired by the ‘proto-cybernetic' theory of learning and memory dating back to the early 1940's (Craik, 1967) and designed around a feedback process. The model was applied on several occasions (Clymer, 1973; Myers, 1992), including Clymer's application of the ‘mnemon' concept in which the characterization of a visual feature induces an associated memory value resulting from experience. This leads to a system where a given visual input induces a response in a specific set of classifying cells that generates a command to attack (i.e., a predatory response) and is further summated to produce an attack ‘strength' (*sensu* Maldonado, 1963c). Conversely, a retreat command is generated by opposing inputs, and their relative strengths are combined to determine the final attack/retreat response (Clymer, 1973). Similarly, another cybernetic circuit was created by Myers (1992) based on octopus' mnemon and neural networks (review in Marini et al., 2017).

As cephalopods are quite distant from, and not as well-characterized as the more familiar, systematically investigated vertebrate models for cognition, exploring the possibility of consciousness in this group of animals necessarily prompts us to ask: how much are they really like the higher vertebrates and to what extent can they be described in terms we readily apply to ourselves, rather than *via* a more mechanistic account?

An insight by Premack and Woodruff is particularly salient here:

´As to the mental states the chimpanzee may infer, consider those inferred by our own species, for example, *purpose or intention*, as well as *knowledge, belief*, *thinking, doubt, guessing, pretending, liking*, and so forthˇ (Premack and Woodruff, 1978, p. 515).

Premack (1988) enumerated this consideration more explicitly by clarifying that the initial question for Woodruff and himself was whether apes “*do what humans do”* and therefore if attributing states of mind to individuals of another species might enable us to predict and explain the behavior of that species (Premack, 1988; see also Emery and Clayton, 2009). The fact that we have only a vague idea of how animals communicate adds another twist to the critical Kantian test (Griffin, 1976). A further complication is that inferences about the possibility that an animal has consciousness inevitably direct or (more often) indirect inferences about our relation to that animal.

In addition, confronting the mental status of a particular animal may summon consideration of the moral status of that animal, its suitability as a model for cognition and behavior, and the acceptability of its use as a commodity or as an experimental substitute for a more ethically ‘indispensable' organism. This last argument is now particularly relevant for cephalopods, considering their inclusion in EU Directive 2010/63 (Smith et al., 2013; Fiorito et al., 2014; Di Cristina et al., 2015) on the grounds of the public perception of these animals and their presumed ability to feel pain and suffering (EFSA Panel, 2005; Smith et al., 2013; Di Cristina et al., 2015). The fact that they are considered to have a degree of sentience (Birch et al., 2021) and may well be capable of at least some form of sensory consciousness would likely be a step forward in defining the parameters of future cephalopod research, including, but not limited to, investigations of behavior and cognition.

### Big-Brained Invertebrates That Engage With a Temporally and Spatially Variable Environment

In a popular essay published more than 30 years ago, Allan Wilson suggested that in vertebrates, there is ´…an autocatalytic process mediated by the brain: the bigger the brain, the greater the power of the species to evolve biologicallyˇ (Wilson, 1985, p. 157). Taking into account increases in genome size, relative brain size, and the number and complexity of neural cell types, Wilson argued that accelerated rates of morphological change in vertebrates over the course of evolution reflected a trend toward increasing complexity of behavioral abilities. In other words, species that had evolved higher numbers and a greater diversity of brain cells and connections were also those which had undergone an increased degree of organization and elaboration of behavioral repertoires. These species were thus able to cope better with environmental changes, accelerating their evolution by adapting more quickly than species in which a lower degree of complexity was achieved. He also suggested that: ´…culturally driven evolution is by no means confined to humans. Imitative learning occurs in many species having brains that are relatively large in relation to body size …. [It] may also occur in some fishes, squids and insects, although it has not yet been demonstrated in them.ˇ (Wilson, 1985, p. 156).

Testing Wilson's Behavioral Drive Hypothesis in cephalopods remains an attractive and intriguing idea (Borrelli, 2007). Such an approach should necessarily incorporate the relationship between the nervous system and the ecology in which it is embedded (e.g., environment and lifestyle/habits; Ponte et al., 2021). It should also consider the computational capacity of the brain - not simply its size. An increase in computational power may have occurred during cephalopod evolution, considering, for example, the reduction in the size of nerve cells between the appearance of squids (i.e., the giant axon) and the later emergence of octopuses (e.g., Young, 1963; Nixon and Young, 2003). Octopus lifestyle must also be taken into account, for example the fact that most species are solitary-living and thus considered to be asocial. Of course, this is not the case for all octopus or cuttlefish species, nor does it appear to be generally true of squid species. Nevertheless, most species of cephalopods have historically been considered asocial animals in the sense that they don't establish or maintain familial relationships and are relatively short-lived (in contrast to the social mammals). Still, this overarching generalization has often been contradicted by both observation and experimental studies (Fiorito and Scotto, 1992; Fiorito, 1993; Huang and Chiao, 2013; Tomita and Aoki, 2014) as well as recent accounts (e.g., Godfrey-Smith and Lawrence, 2012; Amodio and Fiorito, 2013; Guerra et al., 2014; Scheel et al., 2016).

The foregoing prompts some important questions. As a largely asocial animal, why would the octopus have developed the capacity to learn from conspecifics (Fiorito and Scotto, 1992)? Why would a specific population of octopuses travel a fairly significant distance in order to procure coconuts to use as nests (Finn et al., 2009)? And finally, why do the actions of these animals appear intentional to such an extent that their interpretation can confound even the most experienced and least anthropocentric of observers? To this last question, a light rejoinder may have been provided by Buytendijk (1933), who stated that this putative intentionality is conveyed because octopuses give the impression of staring back - or looking you directly in the eye - such that they readily seem to cast their spell on the behavioral scientist.

## Identifying Possible Neural Substrates for Consciousness in Cephalopods

The central brains of cephalopods have unusual features that distinguish them from the nervous systems of other molluscs (see review by Ponte et al., 2021). Among these, the most relevant are:

- The highest degree of centralization among invertebrates (insects excluded), partly due to the shortening of connectives.
- The compact size of neurons acting as local interneurons (e.g., nuclear diameters of 3–5 μm), allowing for a relatively greater cell density.
- The reported absence of somatotopy in these animals, except in the chromatophore lobes, and tract-level representations of the labial nerves, buccal lobe, visceral centers, and funnel nerves (Young, 1965a, 1967, 1971). A recent study provides evidence of marked somatotopy at the level of the basal lobe, where a defined topographical transform from the optic lobes has been identified in squids (Chung et al., 2020); this observation seems to parallel the case of insect and vertebrate brains, in which somatotopy is fairly ubiquitous.
- The presence of a blood-brain barrier, a unique property not found in other molluscs (Abbott and Pichon, 1987; for review see also Dunton et al., 2021).
- Compound field potentials, similar to those recorded in vertebrate brains (e.g., Bullock and Budelmann, 1991; for review see Brown and Piscopo, 2013).
- An elevated efferent innervation of sensory receptors (e.g., the retina and equilibrium receptor organs, among others).
- The presence of peripheral first order afferent neurons (see: Young, 1971, 1991; Brown and Piscopo, 2013).
- A large variety of putative neurotransmitters and neuromodulators (review in Messenger, 1996; Ponte, 2012; Ponte and Fiorito, 2015).

During its evolution, the cephalopod brain achieved maximum aggregation and centralization of neural masses through fusion of the supra– and suboesophageal regions, which came to be enclosed in a cartilaginous cranium along with the expansion of two large optic lobes extending laterally from the supraoesophageal mass (directly behind the eyes). The most radical shift in the gross neural organization of the cephalopods resulted from a change in position and relative volume of the different areas of the nervous system that occurred with the addition or loss of ganglia. The accretion of fused ganglia ultimately yielded a central brain subdivided into a variable number of lobes (depending on species), ranging from 12 in the *Nautilus* to 24 in octopods (excluding the optic lobes). Notably, the central nervous system varies markedly across different cephalopod genera, with grades of neural complexity that parallel the density and complexity of sensory inputs received and the diversity of behaviors controlled and exhibited (Young, 1977a; Maddock and Young, 1987; Budelmann, 1995).

The greatest degree of nervous system centralization among cephalopods is found in the Octopodiformes, and is achieved by the shortening of the pathways connecting the superior buccal and brachial lobes (Nixon and Young, 2003). At the opposite end of the spectrum is the central nervous system of *Nautilus*, with three broad “bands” joining laterally (one dorsal and two ventral to the esophagus; Owen, 1832; Young, 1965b).

Overall, the octopod brain is more centralized than the decapod brain, in which brachial and pedal lobes are fused and the superior buccal lobe is united with the inferior frontal lobes. In addition, the brachial and pedal lobes of octopods, as well as their inferior frontal lobe system, are larger, reflecting the sophisticated use of their arms and highly elaborated chemo-tactile sensory processing and learning. Decapods, in contrast, have larger basal lobes and a simpler inferior frontal lobe system.

As enumerated by Ponte et al. (2021), different cephalopod brains manifest as taxon-specific ‘cerebrotypes' akin to the specific types of brain architectures observed in the vertebrates. The significant quantitative differences between the brains of different cephalopod species reflect variations in habitat (in addition to other physical/environmental conditions). In the great majority of cases, the clusters of identified cerebrotypes correlate with similar ecological and/or behavioral constellations across different cephalopod species (Ponte et al., 2021). Within a total of 52 cephalopod species for which the set of data resulted complete, Ponte and coworkers recognized 10 distinctive groups of species, revealing both differences and close analogies. The overall topology of the relationships among species supports Young's perspective (Young, 1977a) and the working hypothesis that analyses combining relative brain size and life strategies can provide a robust basis for assumptions regarding the selective pressures and adaptations that drove cephalopod evolution. The analysis of cephalopod cerebrotypes (Ponte et al., 2021) highlights a large variation in the relative proportions of brain lobes within the decapods, as well as notable differences in the vertical lobe system when compared to that of the octopods. In fact, *O. vulgaris* presents a vertical lobe made up of *five* folded lobules that produce an overall volume reduction of the structure increasing the surface area and the corresponding number of cells in the lobe. This organization also results in reduction of the neuropilar space, minimization of the length of connections, increase in overall connectivity and computational abilities, akin to that observed in the higher vertebrates (Young, 1963, 1991, 1995; Shigeno et al., 2018). The opposite is true for cuttlefish and squid, where there is no observable folding of the surface of the vertical lobe, the estimated number of cells is much lower, and a correspondingly larger neuropil is found (Ponte et al., 2021).

A close relationship between cerebrotypes and lifestyles in cephalopods has thus been observed, supporting the idea that taxa evolved different sensory and cognitive strategies to cope with the differential demands of life in the ocean (Packard, 1972; Amodio et al., 2019; Ponte et al., 2021; Schnell et al., 2021c). Such complexity and diversity evoke comparisons to similar adaptations found among vertebrates. Taken together, these data support the idea that the appearance of cephalopod cerebrotypes reflect: (i) phylogenetic relationships (e.g., closely related species are likely to have a similar brain composition); (ii) similar developmental trajectories across different species (i.e., paralarvae vs. miniature adults at hatching) and constraints that influence brain organization and function; (iii) ecologically driven behavior which has led to the occupation of similar niches by species that possess similar brain architectures and faculties.

Though certainly noteworthy, the diversity of cerebrotypes is not the sole indicator of cephalopod brain complexity. In a recent review, Shigeno et al.and colleagues sought to establish structural and functional analogies to aspects of the vertebrate brain in the cephalopod nervous system. They undertook an analysis of the sensory, motor, and neurosecretory centers observed in cephalopod brains and attempted to identify ´similarities to the cerebral cortex, thalamus, basal ganglia, midbrain, cerebellum, hypothalamus, brain stem, and spinal cord of vertebratesˇ (Shigeno et al., 2018; see also Table 1 therein). The cephalopod cerebral cord can be considered analogous to the vertebrate forebrain and midbrain, while the pedal and palliovisceral cords are comparable to the vertebrate spinal cord and hindbrain. Evidence for other functional analogs of vertebrate brain features is steadily accumulating. Some examples are discussed below.

First, the existence of a functional analog of the hypothalamus is supported by the presence of neurosecretory cells in different lobes of the cephalopod brain. In vertebrates, the hypothalamus contains a population of neurosecretory cells, among other cell types (Butler and Hodos, 2005). Their evolutionary origins are believed to trace back to a common bilaterian ancestor, perhaps even a pre-bilaterian animal such as a cnidarian (Tessmar-Raible, 2007; Tessmar-Raible et al., 2007). In cephalopods, neurosecretory cells are found mainly in the buccal and sub-pedunculate lobes, as well as in some regions of the dorsal basal lobes, structures which all belong to the supra-esophageal mass (Young, 1970). Other areas reveal potential neurosecretory activity (i.e., sub-buccal and sub-pedunculate, optic gland, the neurovenous tissue of the vena cava; Bogoraze and Cazal, 1946; Barber, 1967; Young, 1970). Some of these regions are candidates for pituitary-hypothalamus analogs in the cephalopod brain, also presenting a subset of neurons containing molecules that are abundant in the hypothalamus, including GnRH and the vasopressin orthologs octopressin and cephalotocin (for review see Shigeno et al., 2018).

Second, the presence of higher sensory centers analogous to the thalamus has recently been proposed (Shigeno et al., 2018). The thalamus is the sensory relay center through which the majority of sensory inputs (excluding olfactory afferents) are directed to the mammalian cerebral cortex or non-mammalian vertebrate pallium (Swanson, 2007). The thalamus acts as a gatekeeper to the cortex and plays a key role in the perception of pain and, of particular note here, the generation of conscious states (Schiff, 2008; Rajneesh and Bolash, 2018; Redinbaugh et al., 2020). The cephalopod dorsal basal- and sub-vertical lobes are considered as candidate analogs of the vertebrate thalamus, as both receive numerous input fibers from the entire body *via* direct and indirect pathways from the sub-esophageal mass, thus acting together as a relay center for the outermost (i.e., cortically disposed) frontal and vertical lobes (Young, 1971). Although at least 10 major tracts originating from and/or terminating at the two structures have been identified in *O. vulgaris* (Young, 1971), to the best of our knowledge no estimation of the number of neural fibers comprising these tracts is available (but see Plän, 1987). Based on its dense connectivity, the dorsal basal lobe has also been proposed as a higher/intermediate motor center.

Furthermore, as discussed by Shigeno et al. (2018), the inferior frontal lobe appears to be another interesting candidate for sensory-motor integration, as a processing center for chemotactile information originating from lower centers (i.e., suckers on the arms), just as the olfactory cortex processes information from the olfactory receptors in vertebrates. Similar to its putative vertebrate counterpart, the inferior frontal lobe is part of the distributed neural matrix involved in learning and memory recall (the so-called chemo-tactile memory system; Young, 1991, 1995). Homologous structures have been identified in the brains of other cephalopods, and future efforts to uncover differences (if any) in the connectivity of the central neural structures of decapods and octopus may provide further insight.

Third, analogs of the vertebrate basal ganglia may be found in the higher motor centers of coleoid cephalopods (Young, 1971, 1977b). In particular, the anterior basal lobes (e.g., supra-esophageal mass) seem to exhibit analogous organization and function. Analysis of their neural connectivity, together with lesion experiments, support such an analogy (Chichery and Chichery, 1987; Gleadall, 1990). Considering their relative location, principal/major connectivity, functional organization (e.g., similarly hierarchical, progressing from motor pattern learning to central pattern controllers, initiators, generators, and motor neuron pools), these lobes are surmised to be plausible functional analogs of their vertebrate counterparts (Shigeno et al., 2018). Such higher motor centers receive sensory inputs and produce responses which, passing through the ‘lower' parts of the central nervous system, are able to regulate posture, orientation, breathing, autonomic control of the viscera, and also habit formation (Shigeno et al., 2018). Analogs of vertebrate basal ganglia and their connections have been identified in different bilaterians (e.g., insects, annelids, and other protostomes) and seem to correspond to the basal lobe systems of cephalopods. However, functional analogies of such structures across taxa are not certain and each motor center has evolved specializations to meet the demands of a specific animal lineage, resulting in different body plans, locomotor systems and lifestyles across these taxa (Shigeno et al., 2018).

Though cortical structures are indeed fundamental for producing conscious states in mammals, subcortical areas are also essential, as they afford the integration of incoming signals into unified percepts and, ultimately, complex motor actions (Afrasiabi et al., 2021).

Given our limited knowledge of the function of the cephalopod basal lobes, as well as insufficiently supported claims regarding the existence of central pattern generators in these animals, we can only encourage further research in this direction.

Fourth and last, we focus on the associative (or auxiliary) centers of cephalopod brain as possible analogs of the vertebrate pallium or mammalian cerebral cortex.

In some cephalopods (e.g., *S. officinalis* and *O. vulgaris*) experimental evidence for sleep (Brown et al., 2006; Meisel et al., 2011; Frank et al., 2012; Iglesias et al., 2019; Medeiros et al., 2021), decision-making (see for example: Maldonado, 1963b, 1965; Carls-Diamante, 2017; Marini et al., 2017; Mather and Dickel, 2017), discrimination learning (for review see: Sanders, 1975; Boal, 1996; Marini et al., 2017), and structural and behavioral lateralization (Jozet-Alves et al., 2012a,b; Schnell et al., 2016a, 2018; Frasnelli et al., 2019) suggests a highly elaborated suite of cognitive faculties. It is not at all inconceivable that such a rich cognitive repertoire would require a neural substrate akin to the mammalian cortex (Edelman and Seth, 2009; Roth, 2015). As reviewed by Shigeno et al. (2018), an extensive series of experiments based on the ablation of different brain areas, followed by behavioral assays, revealed that the frontal and vertical lobe systems (mainly in octopus, but also in cuttlefish) are involved in tactile and visual memory processing. As mentioned earlier, these structures contain large populations of uniquely distributed small interneurons (amacrine cells), parallel-running fibers, and reverberating circuitry across different lobes (Young, 1971, 1979, 1991, 1995). Notably, these are also areas in which synaptic, NMDA-independent long-term potentiation (LTP) has been discovered and characterized (Hochner et al., 2003; Shomrat et al., 2008, 2011; Turchetti-Maia et al., 2017). In addition, these lobe-systems appear to be characterized by heterogeneity of neurochemical identity (Ponte and Fiorito, 2015; Shigeno and Ragsdale, 2015). Further experiments are needed to assess cellular diversity and layered organization (especially of amacrine cells) in the frontal and vertical lobes, though preliminary data, including single cell sequencing, transcriptomes and molecular fingerprints related to learning outcomes (Zarrella, 2011; Zarrella et al., 2015; Manzo, 2021) strongly support this working hypothesis.

## Neurophysiological Dynamics and the Functional Signatures of Conscious States

Electrical activity in cephalopod brain has been assessed through various means and in different contexts (Bullock, 1984; Bullock and Budelmann, 1991; Brown et al., 2006), most recently in the characterization of neural activity (Butler-Struben et al., 2018). In a series of experiments, high-gain bipolar recordings obtained in the dorsal side of cephalopod brain (e.g., vertical lobe) and neighboring structures including the optic lobes (*via* electrodes inserted below the cartilaginous capsule) were able to capture organized electrical activity (Bullock, 1984; Brown et al., 2006). Recordings of brain signals using this methodology, and a similar one adopted by Butler-Struben et al. (2018), revealed periods of relative inactivity, as well as both spontaneous and evoked potentials. Interestingly, spontaneous activity in the areas within the vertical lobe is represented by single spikes and spike trains which are more frequent during rest, indicating a “body off/brain on” type of activation (Brown et al., 2006). Spike trains can last for tens of seconds, with frequencies ranging from about 10 to 40 Hz. The vertical lobe system is involved in learning and memory processing, displaying a vertebrate-like (albeit NMDA-independent) LTP plasticity (for review see Shomrat et al., 2015). Evidence of LTP-like plasticity has also been assessed *in vivo*. The signals recorded in this area in resting animals are believed to be related to memory consolidation, as in the vertebrate case (Shomrat et al., 2008, 2011). Compound potentials can also be evoked robustly in the optic lobes in response to brief flashes of light (Bullock, 1984). Their presence may be related to more basal levels of functional responsiveness of the nervous system to external stimuli.

Notably, the only experimental work involving the exposure of cephalopods to electroconvulsive shock (ECS) was a study by Maldonado (1968, 1969) in *O. vulgaris*. A two-second duration of ECS produced a general paroxysm of muscle contraction, inking, and cessation of breathing, as well as a flattening of the body with a strong adhesion of the suckers to the bottom of the box where the animals were placed. Once the animals were returned to their home tanks, they were initially completely rigid and immobile; breathing resumed shortly thereafter. Normal body posture and locomotor activities were restored within 15 min, and octopuses resumed their normal predatory responses within 2 h following the experiments (Maldonado, 1968, 1969). Interestingly, these studies were employed to assess the impairment of ECS on memory recall, further confirming the existence of sophisticated higher brain function, including the highly conserved biological machinery underlying long term memory.

The foregoing has been interpreted as psychological evidence of compound field potentials in cephalopods that are markedly different than those recorded in other invertebrates. In fact, cephalopod EEGs bear a close resemblance to vertebrate field potential recordings.

As summarized by Amodio and Fiorito (2013), one of the possible constraints on social learning in *O. vulgaris* is the lack of cross-modal integration, i.e., the ability to integrate stimuli from two or more sensory channels (for review see: Borrelli and Fiorito, 2008; Marini et al., 2017; namely, visual- and chemotactile-sensory motor systems). This is especially evident in instances where the solution to a task requires integration of the two modalities, as in the case of certain types of problem solving (Fiorito et al., 1990; Anderson and Mather, 2007; Anderson et al., 2008; Amodio and Fiorito, 2013). However, integration of different sensory channels is clearly demonstrated in foraging activities (Mather, 1991; Mather and O'Dor, 1991) as well as during social recognition, where sight, touch, and olfaction may be part of a multimodal system of information transfer (Partan and Marler, 2005; for examples in octopus see: Tricarico et al., 2011, 2014). Thus, synchronous use of different modalities (i.e., multimodality, Rowe and Guilford, 1999) has the clear advantage of improving detection, recognition, discrimination, and memorization of signals by the receivers, as recently shown in cuttlefish and octopus (Scheel et al., 2016; Schnell et al., 2016b).

Notably, Billard et al. (2020a) demonstrated the ability of cuttlefish to discriminate between and integrate two sensory modalities. Young concluded that complete integration (e.g., transfer) between two (visual and tactile information) systems occurred only at the effector level. However, Allen et al. (1986) showed that a limited degree of cross-modality does exist and the two sensory-motor systems may effectively integrate within higher neural centers: a finding recently supported by behavioral evidence provided by Kawashima et al. (2021).

Neurophysiological investigations have confirmed the view that cuttlefish and octopus evolved neural networks and synaptic plasticity paralleling the classic cellular basis of learning in mammals, i.e., LTP (Hochner et al., 2003; Shomrat et al., 2008, 2011; Hochner and Shomrat, 2013; Turchetti-Maia et al., 2017). However, in terms of architecture and physiological connectivity, the neural substrates for learning and memory in cephalopods evolved in a manner radically different than that of mammalian system (Shigeno et al., 2015), though functional properties analogous to those of mammalian cortical structures still emerged (e.g., limbic lobe as suggested by Young, 1995; Shigeno et al., 2018). These structures - together constituting the vertical lobe system - are characterized by large populations of small nerve cells (e.g., amacrine cells) acting as interneurons which create highly redundant connections working *via en passant* innervations. This feature confers the octopus brain with the ability to create large-capacity memory associations (for review see for example: Sanders, 1975; Young, 1991; Shomrat et al., 2011, 2015; Hochner and Shomrat, 2013; Ponte and Fiorito, 2015). The complexity of neural circuitry is complemented by the rich diversity of neural cell types (e.g., Ponte, 2012; Ponte and Fiorito, 2015; Shigeno and Ragsdale, 2015; Deryckere et al., 2021), with a broad and specific differentiation among areas largely dominated by acetylcholine, catecholamines (dopamine and noradrenaline), indolamines (histamine, 5-HT), octopamine, purines, amino acids, nitric oxide, substance P, somatostatin, FMRF-amide, and other peptides which orchestrate responses at the level of the central and peripheral nervous systems, sensory organs, and viscera of cephalopods (Messenger, 1996). As reviewed by Ponte and Fiorito (2015), only limited regional differences among different neuromodulators appear to exist, and definite boundaries and/or mixing of cellular types have not been identified yet. Moreover, the complex distribution of different cell types in cephalopod brains is far from being characterized in any detail (Ponte, 2012; Ponte and Fiorito, 2015).

The various forms of learning and memory exhibited by cephalopods, the richness and flexibility of their behavioral repertoire (Borrelli and Fiorito, 2008; Marini et al., 2017; Hanlon and Messenger, 2018), and the unique adaptations and operating principles of the neural circuitry underlying their behavioral responses (Hochner et al., 2006; Shomrat et al., 2008, 2011, 2015; Hochner, 2012; Turchetti-Maia et al., 2017; Shigeno et al., 2018) should plausibly be considered markers for the presence of primary consciousness as proposed by Mather (2008).

## Concluding Remarks

The sophisticated behavioral repertoire and cognitive abilities of cephalopod molluscs (Godfrey-Smith and Lawrence, 2012; Amodio and Fiorito, 2013; Tricarico et al., 2014; Scheel et al., 2016) strongly suggest the presence of conscious states in these animals, as further enunciated during the recent well-articulated debate attending the notion of cephalopod ‘mind' (see Mather, 2019)^1^ which included contributions from philosophers and professionals from artistic and cultural domains. While discussion surrounding the attribution of consciousness in cephalopods is still ongoing, the growing body of evidence that, at the very least, it would be prudent to apply the precautionary principle, as implied by the thrust of the present work.

The extraordinary behavioral and cognitive features that cephalopods possess (Godfrey-Smith, 2013, 2016; Marini et al., 2017; Hanlon and Messenger, 2018; Gutnick et al., 2021) have long attracted the public's imagination (e.g., Nakajima, 2018; Nakajima et al., 2018; Holden-Dye et al., 2019). When we consider the neural hallmarks of consciousness (Edelman et al., 2005; Seth et al., 2005; Edelman and Seth, 2009; Edelman, 2011), we must take into account morphological and functional analogies (Young, 1991, 1995; Edelman and Seth, 2009; Albertin et al., 2015; Shigeno et al., 2015; see also Shigeno et al., 2018) which reinforce the argument that nature often achieves the same goals across phylogeny in a number of different ways, some of which may accord with current anatomical and physiological views of how the neural systems underlying complex behavior actually works (see, e.g., Rankin, 2004). It may be useful to recall the argument for biological convergence that was made by Edelman et al. (Edelman et al., 2005; Seth et al., 2005; see also: Edelman and Seth, 2009; Boly et al., 2013) as part of a synthetic approach to the study of animal consciousness. Those Authors argued that, in the assessment of possible conscious states in non-human species, a comparative examination of neuroanatomical, neurophysiological, and behavioral properties and correlates using the human case as a kind of reference standard could provide a way forward. Entertaining the possibility that the phylogeny of consciousness might include some invertebrate lines, they further posited that, even in the absence of neuroanatomy that is structurally *homologous* to that of vertebrates, it is possible that some invertebrates evolved aspects of brain architecture that are functionally *analogous* to neural structures and circuits critical to instantiating conscious states in vertebrates. The fact that invertebrate nervous system do not possess anything that *looks* like cortex, hippocampus, or thalamus does not mean - as we have seen above - that cephalopods are not equipped with specialized structures and circuitry that support *similar functions*, i.e., working and episodic-like memory, storage, and retrieval (or recall) akin to those faculties supported by cortex and hippocampus, as well as recursive - or reentrant - relays that link perception and memory in a manner similar to that afforded by the vertebrate thalamus (see Edelman, 1987, 1989).

In addition to indirect circumstantial evidence of consciousness in cephalopods provided by the outstanding flexibility of their behavioral repertoire, and their relatively complex and specialized neural structures instantiating circuitry resembling that found in vertebrates, the likelihood of consciousness in these invertebrates is supported by more reliable objective, basic neural correlates, such as EEG-like signatures and evoked compound potentials. In acknowledging these facts, the Cambridge Declaration on Consciousness^2^ recognized cephalopods as animals whose neurobiological structures are complex enough to support conscious states. Furthermore, Directive 2010/63/EU has included cephalopods as the sole species among invertebrates listed among the animals whose welfare should be protected for scientific research (Smith et al., 2013; Fiorito et al., 2014, 2015).

Birch et al. suggest that animal consciousness could be conceptualized whitin a broader framework consisting of five dimensions that do not force species into hierarchical positions of higher versus lower levels of consciousness, but rather consider species within their own space: a paradigm that helps us better understand their unique abilities and cognitive profiles without imposing meaningless comparisons (Birch et al., 2020). After all, we cannot expect an octopus to experience the world in the same way that we do. Our sensorimotor systems and the environments we inhabit are radically different and our evolutionary histories are quite divergent. As reviewed above and summarized in Table 1, the five dimensions (Birch et al., 2020) and the hallmarks of consciousness as possible counterparts (Edelman et al., 2005; Seth et al., 2005; Edelman and Seth, 2009) together incorporate perceptual and evaluative richness, integration at both a point in time and over time, and self-awareness (though the distinction between the latter as a higher-order form of consciousness and primary, or sensory, consciousness should be noted).

P-richness refers to the different level of detail with which animals consciously perceive aspects of their environment. Of course, as noted above, this varies according to the sensory systems with which each species is endowed (e.g., chemical-tactile, visual, and auditory). Cephalopods appear to possess a large p-richness in chemo-tactile and visual discrimination (review in: Marini et al., 2017; Mather, 2021b) and are able to retain episodic-like memories (e.g., Pronk et al., 2010; Jozet-Alves et al., 2013).

E-richness refers to the differential affective experience of animals in relation to particular stimuli, and thus to the ability to detect negative or positive valence (in cephalopods see for example: Maldonado, 1963b, 1965; Darmaillacq et al., 2004), which are of course determined by different species- and age-specific physiological needs and motivations. Cephalopods are also likely to have good e-richness, as there is accumulating evidence suggesting the presence of nociception and pain in these animals (Crook et al., 2011, 2013; Alupay et al., 2014; Oshima et al., 2016; Crook, 2021). Unity and Temporality are closely related to how animals subjectively perceive their environments in relation to time and whether they are able to remember, retain, and retrieve information over time (see discussion above). Selfhood refers to an animal's ability to distinguish itself from the outside world and from others (e.g., mirror test).

As summarized in Table 1, sensory-motor communication in the brain of multiple sensorial inputs (p-richness) is the backbone upon which the unity of time-coding, self-awareness, arousal, and motivation are instantiated. In cephalopods the mechanisms of attention and decision making, modulated by D1 or D2 neuronal types in the mammalian striatum, are still unclear. However, the analogies with the mammalian basal ganglia mentioned above, as well as the existence of an intricate dopaminergic (and octopaminergic) network with spatial distribution in specific brain areas (Ponte, 2012; Ponte and Fiorito, 2015) are also indicators of e-richness in cephalopods. Further investigations of possible cephalopod analogs of the cortico-basal ganglia pathways and basal ganglia-thalamic neural pathways will be required to experimentally advance our overview. Gene editing, as recently promoted in cephalopods (Crawford et al., 2020; Steele, 2020) may also help over this challenging avenue.

In mammals, the combination of connectivity-based optogenetic tagging and psychophysical approaches has been pivotal for revealing how interactions between the thalamus and cortex control the sensory and limbic processing that underlies higher cognitive functions (Halassa et al., 2014). Optogenetic studies in mice have allowed the identification of intricate neural networks, possibly contributing to mechanisms of consciousness, including pathways originating from the striatum that inhibit the thalamic reticular nucleus and participate in the regulation of arousal, decision making and states of consciousness (Halassa et al., 2014; Halassa and Kastner, 2017; Schmitt et al., 2017). Optogenetic approaches are in their early infancy in cephalopods, but their potential has been recently exploited with success (Reiter et al., 2018; Reiter and Laurent, 2020). We are convinced that further studies will benefit from an integration of approaches.

Though based on incomplete behavioral, morphological, and physiological findings (thus, considering the precautionary principle; EFSA Panel, 2005), cephalopods have been included in Directive 2010/63/EU as the only invertebrates among the so-called laboratory animals to be protected in scientific research. Originally adopted within the context of environmental law, the ‘precautionary principle' is based on the idea that in cases of threat of actual or potential irreversible damage to the environment, the lack of complete scientific evidences should not be employed as a reason for postponing measures to be taken in order to avoid or minimize the risks (Cameron and Abouchar, 1991; Pinto-Bazurco, 2020). In respect to animals and their welfare, the same principle has been adopted (EFSA Panel, 2005; Andrews, 2011) even employing sentience (Birch, 2017) and consciousness (Bradshaw, 1998; Dawkins, 2017) as justifications. In the words of Bradshaw ´Applying this principle [i.e., precautionary] to the issue of animal consciousness, the following rule is formulated: assume animals do have consciousness in case they do; if they do not it does not matterˇ (1998, p. 108).

It is now evident that adopting a multidimensional approach has completely changed our perspective on animal consciousness and has made us realize that we may have been asking the wrong question, namely “is this species more conscious than that one?,” when the more relevant question should be: “how is the individual experience of this species different from that one?”

The five dimensions enumerated by Birch et al. (2020) are, to some extent, included in the definition of what could be considered the ‘anteroom' of consciousness in animals, namely sentience. According to Broom (2014), a sentient being has at least one of the following abilities: (i) evaluation of the actions of others in relation to itself (e.g., the capacity to form relationships); (ii) the capacity to remember some of one's own actions and their consequences (e.g., cognitive ability); (iii) the ability to assess risks and benefits (e.g., decision-making); (iv) possession of some degree of awareness (e.g., consciousness); (v) the ability to experiencing negative or positive affective states (e.g., the influence of others' states).

Based on the available evidence - reviewed in the present work - we believe that cephalopods are sentient animals in terms of all five capacities summarized above. It will be both intriguing and enlightening to dissect sentience from the cephalopod perspective, based on knowledge accumulated over several decades, as well as on recently gathered evidence and arguments that support the invocation of EFSA guidelines for the inclusion of this taxon in the list of species regulated by the Directive 2010/63/EU (EFSA Panel, 2005; European Parliament Council of the European Union, 2010). But this is a pursuit best reserved for a different time and venue.

## Data Availability Statement

The original contributions presented in the study are included in the article/supplementary material, further inquiries can be directed to the corresponding author.

## Author Contributions

GP, CC, and GF conceived this work. DE contributed to writing together with EP and PI. All authors discussed the content, text and contributed to writing, commented on the manuscript at all stages, and read and approved the submitted manuscript.

## Funding

This work has been supported by the Stazione Zoologica Anton Dohrn and Association for Cephalopod Research CephRes.

## Conflict of Interest

The authors declare that the research was conducted in the absence of any commercial or financial relationships that could be construed as a potential conflict of interest.

## Publisher's Note

All claims expressed in this article are solely those of the authors and do not necessarily represent those of their affiliated organizations, or those of the publisher, the editors and the reviewers. Any product that may be evaluated in this article, or claim that may be made by its manufacturer, is not guaranteed or endorsed by the publisher.

## Footnotes

^1^See also the article thread available at https://www.wellbeingintlstudiesrepository.org/animsent/vol4/iss26/1/

^2^ https://web.archive.org/web/20131109230457/http://fcmconference.org/img/CambridgeDeclarationOnConsciousness.pdf
